# Selective inhibition of dopamine‐beta‐hydroxylase enhances dopamine release from noradrenergic terminals in the medial prefrontal cortex

**DOI:** 10.1002/brb3.393

**Published:** 2015-09-24

**Authors:** Paola Devoto, Giovanna Flore, Pierluigi Saba, Roberto Frau, Gian L. Gessa

**Affiliations:** ^1^ Department of Biomedical SciencesSection of Neuroscience and Clinical PharmacologyUniversity of CagliariCagliariItaly; ^2^ “Guy Everett Laboratory”University of CagliariCagliariItaly; ^3^ Center of Excellence “Neurobiology of Addiction”University of CagliariCagliariItaly; ^4^ Department of Medical SciencesUniversity of CagliariCagliariItaly; ^5^ National Research CouncilCNR, Institute of NeuroscienceCagliariItaly

**Keywords:** α_2_‐adrenoceptor, cocaine, corelease, microdialysis, nepicastat

## Abstract

**Introduction:**

Disulfiram has been claimed to be useful in cocaine addiction therapy, its efficacy being attributed to dopamine‐beta‐hydroxylase (DBH) inhibition. Our previous results indicate that disulfiram and the selective DBH inhibitor nepicastat increase extracellular dopamine (DA) in the rat medial prefrontal cortex (mPFC), and markedly potentiated cocaine‐induced increase. Concomitantly, in rats with cocaine self‐administration history, cocaine‐seeking behavior induced by drug priming was prevented, probably through overstimulation of D1 receptors due to the DA increase. The present research was aimed at studying the neurochemical mechanisms originating the enhanced DA release.

**Methods:**

Noradrenergic system ablation was attained by intracerebroventricular (i.c.v.) administration of the neurotoxin anti‐DBH‐saporin (aDBH‐sap). DA, noradrenaline (NA), and DOPAC were assessed by HPLC after ex vivo tissue extraction or in vivo microdialysis. Control and denervated rats were subjected to microdialysis in the mPFC and caudate nucleus to evaluate the effect of nepicastat–cocaine combination on extracellular DA levels and their regulation by α_2_‐adrenoceptors.

**Results:**

Fifteen days after neurotoxin or its vehicle administration, tissue and extracellular NA were reduced to less than 2% the control value, while extracellular DA was increased by approximately 100%. In control rats, nepicastat given alone and in combination with cocaine increased extracellular DA by about 250% and 1100%, respectively. In denervated rats, nepicastat slightly affected extracellular DA, while in combination with cocaine increased extracellular DA by 250%. No differences were found in the caudate nucleus. Clonidine almost totally reversed the extracellular DA elevation produced by nepicastat–cocaine combination, while it was ineffective in denervated rats.

**Conclusions:**

This research shows that the increase of extracellular DA produced by nepicastat alone or in combination with cocaine was prevented by noradrenergic denervation. The results indicate that nepicastat enhances DA release from noradrenergic terminals supposedly by removing NA from α_2_‐autoreceptors. In addition to the inhibition of DA uptake, the latter mechanism may explain the synergistic effect of cocaine on nepicastat‐induced DA release.

## Introduction

Dopamine‐beta‐hydroxylase (DBH), the enzyme that converts dopamine (DA) to noradrenaline (NA), is a promising target for pharmacotherapies targeting cocaine (George et al. 2000; Petrakis et al. 2000; Carroll et al. 2004; Kosten et al., 2013), alcohol dependence (Johansson 1992; Colombo et al. 2014), and eating disorders (Zaru et al. 2013; Farci et al. 2015).

Disulfiram, which in addition to aldehyde dehydrogenase (ALDH) (Lipsky et al. 2001) also inhibits DBH (Goldstein et al. 1964; Musacchio et al. 1966), was initially used in patients simultaneously abusing alcohol and cocaine, based on the rationale that it would deter alcohol use and thus eliminate alcohol priming effect on cocaine use (Higgins et al. 1993; Carroll et al. 2000). Subsequently disulfiram was found to be even more effective in reducing the frequency and amount of cocaine use in nonalcoholic cocaine‐dependent patients, suggesting that it directly impacts the behavioral response to cocaine (Hameedi et al. 1995; McCance‐Katz et al. 1998a,b; George et al. 2000; Carroll et al. 2004).

The efficacy of disulfiram in the treatment of cocaine dependence has been attributed to an increase in brain dopamine (DA) resulting from DBH inhibition, which in turn corrects the “hypodopaminergia” present in cocaine‐dependent subjects, purportedly responsible for loss of control and compulsive drug use (Petrakis et al. 2000; Volkow et al. 2009). Alternatively, it has been suggested that excessive DA release following cocaine use after disulfiram treatment may be associated with anxiety and dysphoria, rather than euphoric response, resulting in reduced cocaine use (McCance‐Katz et al. 1998a,b; Kosten et al. 2002).

However, as besides ALDH and DBH, disulfiram also inhibits a series of copper‐containing enzymes and different esterases, including plasma cholinesterase involved in cocaine metabolism (Hameedi et al. 1995; Baker et al. 2007), its mechanism of action in clinical application remains unclear.

Experimental investigations have provided an important contribution toward clarifying this problem. Consistent with clinical results obtained, Schroeder et al. (2010) have shown in rats that disulfiram inhibits cocaine‐primed reinstatement of cocaine‐seeking behavior after extinction, with this effect being reproduced by nepicastat, a selective DBH inhibitor, devoid, unlike disulfiram, of ALDH inhibitory property (Stanley et al. 1997). These authors suggested that DBH inhibitors, by reducing NA formation, would decrease noradrenergic drive onto midbrain dopaminergic neurons, which is essential for cocaine‐induced DA release and consequent reinstatement of cocaine‐seeking behavior (Schank et al. 2006; Gaval‐Cruz and Weinshenker 2009; Schroeder et al. 2010, 2013). Accordingly, the same authors predicted that DBH inhibitors should attenuate dopaminergic firing and cocaine‐induced release in the nucleus accumbens and prefrontal cortex.

However, at variance with these assumptions, empirical evidence from our laboratory indicates that both disulfiram and nepicastat produce, as expected, not only a widespread reduction in tissue NA content and release, but they also increase DA release in the mPFC, an effect markedly potentiated by cocaine. Moreover, both DBH inhibitors were also found to increase, although modestly, DA release in the nucleus accumbens and to not modify cocaine‐induced DA release in this region. It should be highlighted that, to the best of our knowledge, the effect of DBH inhibitors on the firing of meso‐cortico‐limbic dopaminergic neurons has not been tested to date. To explain our results we suggested that DBH inhibitors cause a lack of NA at release‐inhibiting α_2_‐autoreceptors, leading to unrestrained release of DA, substituting for NA, from noradrenergic terminals.

The present study intended to provide direct evidence that DBH inhibitors increase DA release from noradrenergic terminals in the mPFC. To this aim, we verified whether the effect of nepicastat on DA release was modified after selective central noradrenergic denervation produced by an anti‐DBH‐antibody conjugated to the ribosomal toxin saporin (aDBH‐sap) that has the ability, after intraventricular injection, to effectively and selectively destroy the majority of central noradrenergic neurons (Wrenn et al. 1996; Rohde and Basbaum 1998).

## Methods

All experiments were approved by the local Ethical Committee and performed according to the European Communities Council Directive of 24 November 1986 (86/609/EEC) in compliance with the “Principles of Laboratory Animal Care” guidelines. Male Sprague Dawley rats (Harlan Italy, S. Pietro al Natisone, Italy), weighing 175–200 g on arrival, were housed in groups of five per cage for at least 7 days before use, under standard conditions of temperature and humidity and artificial light from 8 am to 8 pm; food and water were available ad libitum. Experiments were conducted from 9 am to 5 pm. A total of 73 animals were used.

Noradrenergic system ablation was achieved by administration of the selective and potent neurotoxin aDBH‐sap. To this aim, rats were deeply anesthetized with Equithesin (containing, per 100 mL, 0.97 g pentobarbital, 2.1 g MgSO_4_, 4.25 g chloral hydrate, 42.8 mL propylene glycol, 11.5 mL 90% ethanol; 5 mL/kg, i.p.) and placed in a Kopf stereotaxic apparatus. The skull was exposed and a hole was drilled directly into the lateral ventricle (AP −1.0, L ± 1.5 from the bregma, V −4.3 from skull, coordinates according to Paxinos and Watson 1997) to allow the administration of immunotoxin (*n* = 35) or vehicle (*n* = 38) solutions. aDBH immunotoxin was diluted in a sterile‐filtered phosphate‐buffered saline (vehicle: 140 mmol/L NaCl, 3 mmol/L KCl, 2 mmol/L KH_2_PO_4_, 10 mmol/L Na_2_HPO_4_, pH 7.4) and injected intracerebroventricularly (i.c.v.) in a volume of 5 *μ*L with a 10 *μ*L syringe operated by a CMA/100 microinjection pump (CMA Microdialysis, Stockholm, Sweden) at a rate of 1 *μ*L/min over 5 min, followed by a 2‐min pause before slowly withdrawing the needle. Injections were randomly distributed into either the left or right lateral ventricles. Control rats received the corresponding volume of vehicle, alone or containing either saporin or a nonspecific mouse IgG conjugated with saporin in equimolar concentrations to the immunotoxin. Preliminary experiments indicated that tissue and extracellular DA and NA values from vehicle‐treated rats were no different from values obtained from rats injected i.c.v. with vehicle containing saporin or IgG‐saporin, or from intact rats. Rats treated with vehicle were therefore used as controls. Rats were given antibiotic therapy (enrofloxacin; Bayer HealthCare, Shawnee Mission, KS) for 5 days and allowed to recover in their home cages for 15–18 days; they were then stereotaxically implanted with vertical microdialysis probes (membrane AN 69‐HF, Hospal‐Dasco, Bologna, Italy; cutoff 40,000 Daltons, 4‐mm active membrane length), in the mPFC (AP +3.0, L ± 0.6, V −6.5 from the bregma, according to the coordinates of Paxinos and Watson 1997), under Equithesin anesthesia. The day after probe implantation, artificial cerebrospinal fluid (147 mmol/L NaCl, 4 mmol/L KCl, 1.5 mmol/L CaCl_2_, 1 mmol/L MgCl_2_, pH 6–6.5) was pumped through the dialysis probes at a constant rate of 1.1 *μ*L/min via a CMA/100 microinjection pump (Carnegie Medicine, Stockholm, Sweden) in freely moving animals, and dialysate samples were collected every 20 min. NA, DA, and DOPAC were simultaneously analyzed by HPLC with electrochemical detection, by HPLC systems equipped with 3.0 × 150 mm C18 (3.5 *μ*) symmetry columns (Waters, Milan, Italy), maintained at 40°C by Series 1100 thermostats (Agilent Technologies, Waldbronn, Germany), and ESA Coulochem II detectors (Chelmford, MA). The mobile phase consisted of 80 mmol/L Na_2_HPO_4_, 0.27 mmol/L EDTA, 0.6 mmol/L sodium octyl sulfate, 8% methanol, 3% acetonitrile, pH 2.8 with H_3_PO_4_, delivered at 0.3 mL/min; the Coulochem analytical cell first electrode was set at +200 mV, the second at −200 mV. Quantification was performed by recording the second electrode signal. Under these conditions, NA and DA detection limits (signal‐to‐noise ratio 3:1) were 0.3 pg per injection on column. In all aDBH‐sap‐lesioned rats, extracellular NA levels were lower than the HPLC detection limit. On completion of testing, rats were sacrificed by Equithesin overdose, the brains removed and sectioned by a cryostat (Leica CM3050 S) in 40‐*μ*m thick coronal slices to verify locations of dialysis probes. No animal was found with errant location of the device.

To evaluate tissue contents of NA, DA, and DOPAC, rats were sacrificed by decapitation (*n* = 18 denervated and 17 control rats); brains were rapidly removed and placed on a brain cutting block maintained on ice. The mPFC was dissected out from 2‐mm slices, immediately frozen on dry ice, and stored at −80°C until processing for catecholamine content. Briefly, tissues were weighed, homogenized by sonication in 0.1 mol/L HClO_4_ (1:20 weight tissue per solvent volume), centrifuged at 10,000*g*, the supernatant filtered using microspin centrifuge tubes (0.22‐*μ*m nylon filter), and directly injected into the HPLC column under analytical conditions as described for microdialysis experiments. Data were expressed as pg neurotransmitter per mg tissue.

### Drugs

Immunotoxin aDBH‐sap, IgG‐saporin, and saporin were purchased from Advanced Targeting System (San Diego, CA). Nepicastat was a gift from Biotie Therapies (Basel, Switzerland), clonidine was purchased from Tocris (Bristol, UK), cocaine hydrochloride from Macfarlan Smith Ltd. (Edinburgh, UK). Nepicastat was used at the dose of 50 mg/kg per mL i.p., dissolved by sonication in DMSO/water (50/50); clonidine (0.15 mg/kg) and cocaine (10 mg/kg) were dissolved in sterile water. All drugs were administered i.p. in a volume of 1 mL/kg weight.

### Statistical analysis

To compare catecholamine levels in denervated and control animals, significances of intergroup differences were calculated using the unpaired two‐tailed Student's *t*‐test with Welch's correction. To evaluate the effect of drug administration versus baseline level, one‐way ANOVA for repeated measure with Bonferroni's post hoc test was applied. To compare the drug effects between the two treatment groups (vehicle vs. aDBH‐sap), repeated measure two‐way ANOVA with Sidak's multiple comparison test as post hoc was applied. Statistical significance was set at *P* < 0.05. Statistical analysis employed Prism 6.0c program (GraphPad Software Inc., San Diego, CA).

## Results

As shown in Tables 1 and 2, the intraventricular administration of aDBH‐sap reduced mPFC tissue and extracellular NA content to less than 2% with respect to control rats, while it failed to modify tissue DA content and increased extracellular DA by about 100%, with respect to control rats. In denervated rats, tissue and extracellular DOPAC levels were modestly reduced and unchanged, respectively, with respect to control rats, indicating that denervation‐induced extracellular DA accumulation is the result of a reduced DA retrieval from extracellular spaces rather than increased DA synthesis and metabolism in dopaminergic neurons.

**Table 1:** Tissue catecholamine and DOPAC content in the medial prefrontal cortex of rats treated with aDBH‐sap or vehicle

Treatment	Noradrenaline	Dopamine	DOPAC
Vehicle	473.6 ± 16.9	98.8 ± 4.7	31.2 ± 1.5
a‐DBH‐sap	5.6 ± 1.3**	85.4 ± 9.6	24.5 ± 2.7*

Values are expressed as pg/mg tissue, and given as the mean ± SEM of 18 rats, sacrificed 16–19 days after the intraventricular infusion of the vehicle or the antidopamine‐*β*‐hydroxylase saporin (aDBH‐sap).

**P < 0.0001 vs. vehicle; *P < 0.05 (two‐tailed, unpaired Student's *t*‐test with Welch correction).

**Table 2:** Extracellular catecholamine and DOPAC levels in the medial prefrontal cortex of rats treated with aDBH‐sap or vehicle

Treatment	Noradrenaline	Dopamine	DOPAC
Vehicle	3.56 ± 0.24	1.50 ± 0.09	186.40 ± 29.60
a‐DBH‐sap	ND^1^	3.22 ± 0.29*	211.12 ± 29.86

Extracellular noradrenaline, dopamine, and DOPAC basal levels in the medial prefrontal cortex of rats injected i.c.v. with antidopamine‐*β*‐hydroxylase saporin (aDBH‐sap) or vehicle. Results are means ± SEM of values obtained from 21 control and 17 lesioned rats, and are expressed as pg per sample.

^1^Noradrenaline levels were lower than the detection limit in all lesioned rats.

*P < 0.0001 vs. vehicle (two‐tailed, Welch‐corrected unpaired Student's *t*‐test).

**Figure 1:**
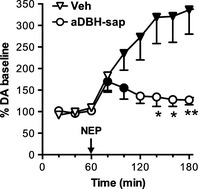
Effect of nepicastat (50 mg/kg, i.p.) on extracellular dopamine levels in the medial prefrontal cortex of control (*n* = 6) and aDBH‐sap treated (*n* = 5) rats. Results are the mean ± SEM expressed as percentage of mean basal level. Arrow indicates the time of nepicastat administration. Black symbols indicate *P* < 0.05 with respect to basal values (one‐way RM‐ANOVA with Bonferroni's test as post hoc); asterisks indicate time points significantly different between the two treatment groups (two‐way RM‐ANOVA, with Sidak's multiple comparisons test as post hoc). *P < 0.05; **P < 0.01.

Consistent with previous observations, in control rats nepicastat, at the dose of 50 mg/kg, increased extracellular DA to about 350% the basal value, with this increase persisting at 2 h after treatment (Fig. 1).

In contrast, in denervated rats nepicastat effect was modest and short lasting: extracellular DA levels increased to 150% the baseline at 20 min and returned to baseline within 60 min (Fig. 1). Statistical analysis by two‐way RM‐ANOVA, with treatment (Vehicle, aDBH) as between‐subject factor and time (using the samples collected after nepicastat treatment) as within‐subject repeated measure factor, indicated a significant effect of treatment (*F*_1,9_ = 5.62; *P* < 0.05) and time (*F*_5,45_ = 4.92; *P* < 0.002). Post hoc Sidak's multiple comparisons test evidenced that nepicastat effect was significantly different between control and lesioned animals at time points 140, 160 (*P* < 0.05), and 180 min (*P* < 0.01).

**Figure 2:**
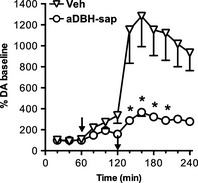
Effect of nepicastat (50 mg/kg, i.p.) and cocaine (10 mg/kg i.p.) coadministration on extracellular dopamine levels in the medial prefrontal cortex of vehicle (*n* = 5) and aDBH‐sap (*n* = 4) treated rats. Results are the mean ± SEM expressed as percentage of mean basal level. The first arrow indicates the time of nepicastat administration, the second indicates cocaine administration. Asterisks indicate significantly different time points between treatment groups (two‐way RM‐ANOVA, with Sidak's multiple comparisons test as post hoc). *P < 0.05.

As Figure 2 shows, in control rats the coadministration of nepicastat and cocaine (50 and 10 mg/kg i.p., respectively) produced a striking 1200% increase of extracellular DA, a much higher increase than the sum of the effect of each compound given alone. In denervated rats the drug combination increased extracellular DA to about 350%, roughly the sum of the effects of cocaine and nepicastat observed in these animals (Fig. 2). Two‐way ANOVA resulted in a significant effect of treatment (*F*_1,7_ = 8.07; *P* < 0.05) and time (*F*_5,35_ = 4.30; *P* < 0.005).

**Figure 3:**
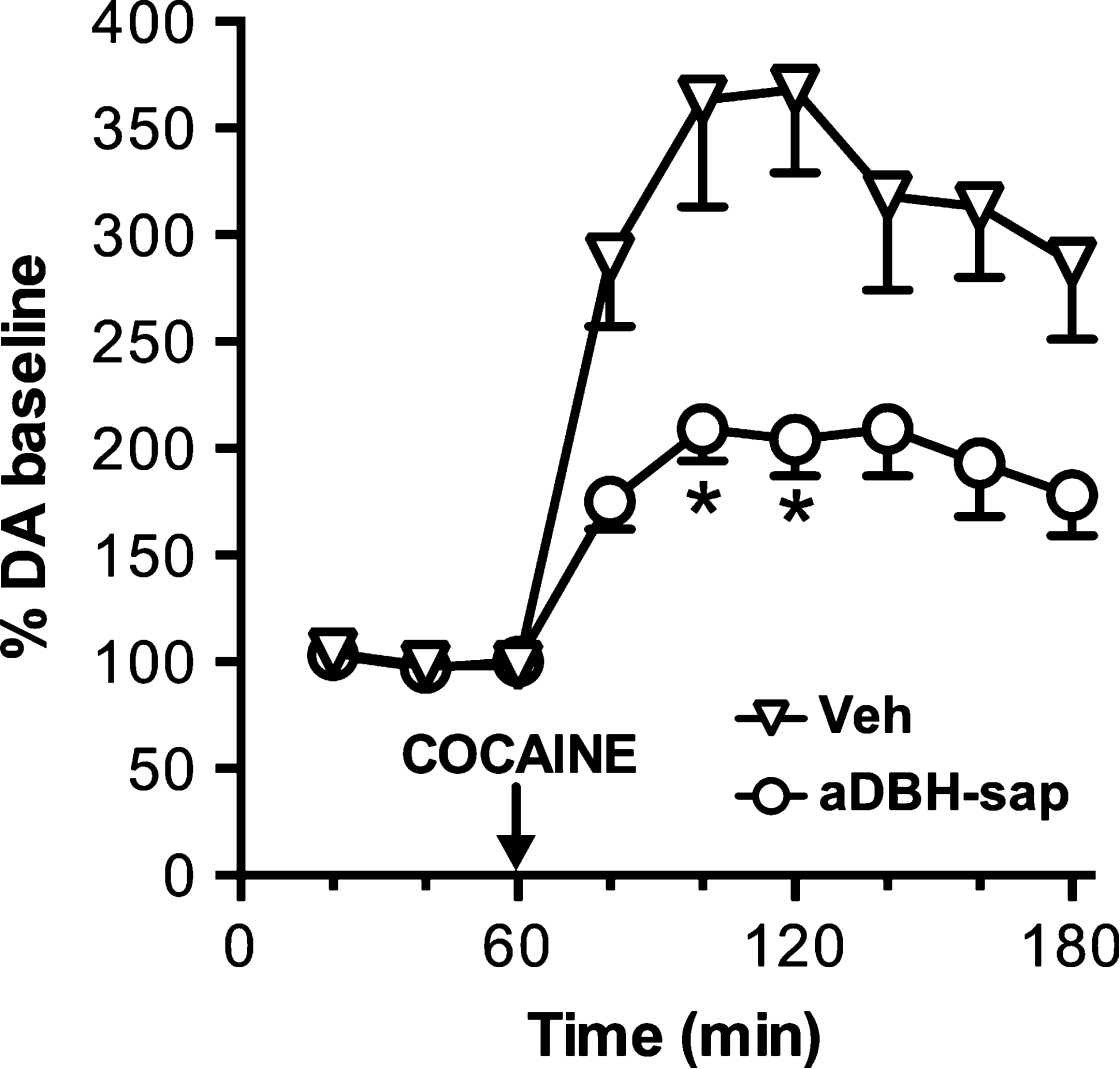
Effect of cocaine (10 mg/kg, i.p.) on extracellular dopamine levels in the medial prefrontal cortex of vehicle (*n* = 6) and aDBH‐sap treated (*n* = 4) rats. Results are the mean ± SEM expressed as percentage of mean basal level. Arrow indicates the time of cocaine administration. Asterisks indicate significantly different time points between the two treatment groups (two‐way RM‐ANOVA, with Sidak's multiple comparisons test as post hoc). *P < 0.05.

As Figure 3 shows, denervation also reduced the effect of cocaine given alone. At the dose of 10 mg/kg, cocaine increased extracellular DA to approximately 350% in control rats, but only to 200% in denervated rats. Statistical analysis by two‐way ANOVA, with treatment (Vehicle, aDBH) as between‐subject factor and time as within‐subject repeated measure factor, indicated a significant effect of treatment (*F*_1,8_ = 7.92; *P* < 0.05) and time (*F*_5,40_ = 3.36; *P* < 0.02).

**Figure 4:**
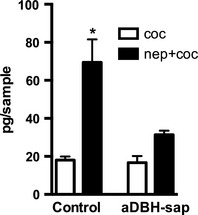
Effect of cocaine, alone or associated with nepicastat, on dopamine output in intact (control) and denervated (aDBH‐sap) rats. Dopamine output is expressed as cumulative amount of dopamine above the baseline, measured during six consecutive dialysate samples after cocaine treatment. Each bar represents the mean ± SEM of 4–6 rats. *P < 0.01 with respect to corresponding column in aDBH‐sap treated rats (one‐way ANOVA, with Sidak's multiple comparisons test as post hoc).

However, since DA basal levels in denervated rats were 100% higher than in control rats, DA increase produced by cocaine in control and denervated rats were recalculated as net of baseline absolute amounts of DA released. As Figure 4 shows, cocaine increased extracellular DA by the same degree in control and denervated rats. In control rats the combination of nepicastat and cocaine produced an increase of 70 pg (cumulative effect during the 2 h collection period after cocaine injection), whereas in denervated rats the increase was of about 30 pg. One‐way ANOVA indicated a very significant treatment effect (*F*_3,14_ = 13.1, *P* < 0.001); the net increase produced by nepicastat–cocaine association resulted to be significantly different between control and denervated animals (Tukey's multiple comparisons test: *P* < 0.001), but nepicastat plus cocaine‐induced increase was not significantly different from cocaine‐induced increase in denervated animals (Tukey's multiple comparisons test: *P* = ns).

**Figure 5:**
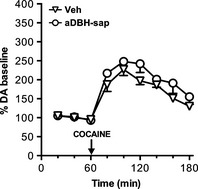
Effect of cocaine (10 mg/kg, i.p.) on extracellular dopamine levels in the caudate nucleus of vehicle (*n* = 5) and aDBH‐sap treated (*n* = 4) rats. Results are the mean ± SEM expressed as percentage of mean basal level. Arrow indicates the time of cocaine administration.

On the other hand, denervation failed to modify the effect of cocaine on extracellular DA in the caudate nucleus, with cocaine increasing extracellular DA to approximately 250% in both intact and denervated rats (Fig. 5). Two‐way ANOVA indicated a highly significant effect of time (*F*_5,35_ = 13.0; *P* < 0.0001), but no significant effect of treatment (*F*_1,7_ = 5.15; *P* > 0.05).

**Figure 6:**
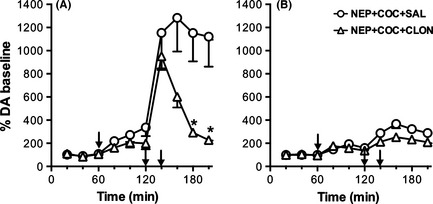
Effect of clonidine (0.15 mg/kg i.p.) on nepicastat plus cocaine administration (50 + 10 mg/kg i.p., respectively), on extracellular dopamine levels in the medial prefrontal cortex of control (A) and aDBH‐lesioned (B) rats. Results are the mean ± SEM expressed as percentage of mean basal level. The first arrow indicates the time of nepicastat administration, while the second indicates cocaine administration. Clonidine was injected 20 min after cocaine (third arrow). Asterisks indicate significantly different time points between the two treatment groups (two‐way RM‐ANOVA, with Sidak's multiple comparisons test as post hoc). *P < 0.05.

Consistent with previous observations, systemic administration of the α_2_‐adrenoceptor agonist clonidine (0.15 mg/kg, i.p.) produced a rapid and complete reversal of the extracellular DA elevation produced by nepicastat–cocaine combination (Fig. 6A). In contrast, in denervated rats treated with nepicastat–cocaine combination, clonidine failed to significantly modify extracellular DA levels (Fig. 6B). Two‐way ANOVA evidenced in both groups a significant effect of treatment (control: *F*_1,4_ = 39.8, *P* < 0.005; lesioned: *F*_1,5_ = 8.13, *P* < 0.05) and time (control: *F*_4,16_ = 19.5, *P* < 0.0001; lesioned: *F*_4,20_ = 9.06, *P* < 0.001). Post hoc Sidak's multiple comparison test indicated as significantly different time points from 180 to 240 for control rats and 220 to 240 for aDBH‐lesioned animals.

## Discussion

Consistent with previous studies showing that the anti‐DBH‐saporin efficiently and selectively destroys the majority of CNS noradrenergic neurons after intraventricular administration (Wrenn et al. 1996; Rohde and Basbaum 1998), we found that the toxin depleted tissue and extracellular NA by almost 98% in the mPFC, but failed to reduce tissue DA, and actually increased extracellular DA by approximately 100% above the level measured in control rats.

DOPAC concentration was reduced in tissue but not in dialysate from lesioned animals, as expected consequence of decreased intracellular DA catabolism due to loss of noradrenergic neurons. These findings are in apparent contrast with our former hypothesis that the majority of extracellular DA in the mPFC originates from noradrenergic terminals (Devoto et al. 2001; Devoto and Flore 2006). However, DA elevation present after denervation may be explained by a loss of NA transporter (NET), the principal mechanism of DA uptake in the mPFC (Carboni et al. 1990; Pozzi et al. 1994; Moron et al. 2002), the possible sprouting of dopaminergic fibers compensating for loss of noradrenergic innervation, and loss of NA at putative release‐inhibiting α_2_‐heteroreceptors on dopaminergic terminals.

A major outcome of this study is the finding that noradrenergic denervation prevented almost completely nepicastat‐induced DA release, and also drastically reduced extracellular DA accumulation produced by cocaine–nepicastat association in the mPFC, but failed to do so in the caudate nucleus, where dopaminergic innervation is prevalent over noradrenergic innervation.

Indeed, in control rats the coadministration of cocaine and nepicastat produced a striking 12‐fold increase of extracellular DA, whereas in denervated rats the increase was 30% that observed in intact rats. These results suggest that the majority of extracellular DA released by nepicastat given alone or in combination with cocaine originates from noradrenergic terminals. This hypothesis may explain why the magnitude of extracellular DA elevation observed following administration of the DBH inhibitors nepicastat or disulfiram was proportional to the ratio of noradrenergic to dopaminergic innervation in the brain region examined, being highest in the mPFC and occipital cortex, modest in the nucleus accumbens and null in the caudate nucleus (Devoto et al. 2012, 2014a).

To explain extracellular DA elevation after DBH inhibitors, we postulated that DBH inhibition causes a lack of NA at release‐inhibiting α_2_‐autoreceptors, leading to unrestrained release of DA, instead of NA, from noradrenergic terminals in the mPFC (Devoto et al. 2012, 2014a). This hypothesis is supported by our previous studies indicating that disulfiram‐induced DA release in the mPFC was reversed by locally infused tetrodotoxin or by the α_2_‐adrenoceptor agonist clonidine, systemically injected or locally infused at nerve terminal level, but was not further augmented by administration of the α_2_‐adrenoceptor antagonist RS 79948, consistent with the hypothesis that autoreceptors were not occupied by NA after DBH inhibition (Devoto et al. 2012). A lack of NA at release‐inhibiting α_2_‐autoreceptors might contribute, together with cocaine‐induced blockade of DA clearance from extracellular spaces, toward the synergistic potentiation of nepicastat effect by cocaine.

Our results do not rule out the possibility that DA released from dopaminergic terminals may contribute to the effect produced by nepicastat. Indeed, α_2_‐adrenoceptor immunoreactivity has been detected in the majority of dopaminergic neurons in the ventral tegmental area (Lee et al. 1998), suggesting the existence of release‐inhibiting α_2_‐heteroreceptors on dopaminergic terminals in the mPFC. However, the contribution of dopaminergic terminals to nepicastat‐induced DA output seems to be lower than that of noradrenergic ones, since denervation, which is expected to eliminate both NET and the presence of NA at α_2_‐adrenoceptors, including α_2_‐heteroreceptors, produced a rather modest increase of extracellular DA when compared with the marked elevation previously observed in intact rats, in which NET and α_2_‐adrenoceptors were pharmacologically inactivated (Devoto et al. 2004).

Moreover, clonidine totally reversed extracellular DA elevation produced by nepicastat–cocaine combination in intact rats, but failed to reduce extracellular DA in denervated rats, consistent with the hypothesis that clonidine acts on α_2_‐autoreceptors rather than α_2_‐heteroreceptors at dopaminergic terminals, although the possibility that denervation might have altered the sensitivity of α_2_‐adrenoceptors to clonidine cannot be dismissed.

Our results provide indirect support to the hypothesis that DA normally released from noradrenergic terminals may account for the major portion of extracellular DA in the mPFC, and for most of extracellular DA in occipital, parietal, and cerebellar cortices, where dopaminergic fibers are scarce or absent (Devoto et al. 2001). Consistent with these considerations, a recent study by Smith and Greene (2012) indicates that noradrenergic fibers in the hippocampus are the origin of DA released by amphetamine, suggesting that DA from noradrenergic terminals in hippocampus plays a key role in learning state and potentially in addictive processes.

Irrespective of the source of DA in the mPFC released by nepicastat and its coadministration with cocaine, the elevation of extracellular DA poses the problem of its physiological significance, specifically the suppressant effect produced on cocaine‐primed reinstatement of cocaine seeking in rats, and its clinical anticocaine effect.

The accepted view that cocaine‐primed reinstatement of drug‐seeking behavior is mediated by DA, possibly acting on D1 receptors (Capriles et al. 2003; Kalivas and McFarland 2003), is not in contrast to our hypothesis that the suppressant effect of DBH inhibitors is due to an excess of DA in the mPFC leading to a supranormal stimulation of D1 receptor signaling in the dorsal m PFC (Devoto et al. 2014b). Indeed we postulated that an optimal level of D1 receptor stimulation is required for reinstatement of drug seeking, while an excessive stimulation leads to suppression of this behavior. This hypothesis is consistent with previous researches that indicate an inverted “U” shaped function for the D1 receptor stimulation of neuronal activity in the mPFC and correlated with cognitive performances (Vijayraghavan et al. 2007).

Further research should clarify whether DA release in the mPFC might mediate the suppressant effect of nepicastat or disulfiram on reinstatement of cocaine seeking triggered by cues and stress other than by cocaine itself (Schroeder et al. 2013). On the other hand, our results signal the possible risk that an excessive release of DA in subjects assuming cocaine after treatment with nepicastat might precipitate psychotic symptoms, as observed following disulfiram–cocaine interaction (Cubells et al. 2000; Mutschler et al. 2009; Grau‐López et al. 2012). These considerations are of considerable relevance as nepicastat is currently undergoing Phase 2 development for cocaine dependence (ClinicalTrials.gov identifier: NCT01704196, A Multi‐Center Trial of Nepicastat for Cocaine Dependence).

## Conflict of Interest

None declared.
